# Drug‐Refractory Dystonia: Benefits of Lamotrigine Therapy in a Case of *SCN2A* Epilepsy‐Dyskinesia Syndrome

**DOI:** 10.1002/mdc3.70842

**Published:** 2026-10-08

**Authors:** Isobel S. Platt, Patrick B. Moloney, Sanjay M. Sisodiya, Thomas T. Warner

**Affiliations:** ^1^ Department of Clinical and Movement Neurosciences University College London London UK; ^2^ Department of Epilepsy UCL Queen Square Institute of Neurology Queen Square London UK; ^3^ Chalfont Centre for Epilepsy Buckinghamshire UK

**Keywords:** gain‐of‐function, encephalopathy, drug treatment, sodium channel blocker

Lamotrigine offers a promising alternative to conventional dystonia treatments in patients with predicted and confirmed gain‐of‐function *SCN2A* mutations.

## Case Report

A 25‐year‐old woman presented from birth with a developmental and epileptic encephalopathy (DEE), with later emergence of episodic involuntary generalized stiffness and posturing of the limbs and trunk.^1^

Developmental history included suspected seizures in utero. This was their mother's only pregnancy, delivered at 42 weeks gestation following labour complicated by meconium aspiration. Postnatally, motor seizures occurred up to five times daily. For the first 7 months, she required hospitalizations for refractory seizures. Medical history included retinal degeneration, scoliosis, laryngomalacia and microcephaly.

Seizures were characterized by left head deviation, right arm elevation and associated limb stiffness. Video electroencephalography (vEEG) at 1 and 5 years confirmed clinical and subclinical seizures with left central epileptiform activity. Brain MRI at 1 year demonstrated left hemispheric white matter changes. Good seizure control was achieved with topiramate and levetiracetam, and she had no generalized seizures after age 8.

Aged 9, next generation sequencing‐based gene panel screening 48 genes associated with severe developmental delay and seizures identified *a* de novo mutation in the gene encoding sodium voltage‐gated channel alpha subunit 2 (*SCN2A*, *OMIM 182390*) (NM_021007.2: c.2619C>G p.(Ile873Met)).^2^ This variant is absent from gnomAD, occurs in a gene with low tolerance for missense variation, and is predicted to be deleterious by multiple in silico tools. The American College of Medical Genetics and Genomics (ACMG) criteria classify this as likely pathogenic and considered causative. Prediction tools indicated 86% likelihood that the *SCN2A* variant causes a gain‐of‐function (GoF) effect, compatible with clinical features of early‐onset seizures and encephalopathy.^3^, ^4^ Whole genome sequencing (WGS) was not performed.

During late teens she developed slowly progressive involuntary movements consisting of mobile dystonic posturing of feet and right upper limb. Exacerbations occurred without altered awareness, characterized by stertorous breathing, marked retrocollis with opisthotonus, spinal hyperlordosis and marked limb posturing. These have been described elsewhere by Papandreou et al.^1^ Episodes occurred up to five times a day, each lasting from minutes to hours, exacerbated during menses. At presentation to the movement disorders service aged 20 she required chloral hydrate daily to reduce episode severity and duration, independent from prescribed medications. She required several admissions for sustained episodes. Repeat EEG captured posturing without clear epileptiform discharges, indicating the events were non‐epileptic (Video 1, Fig. 1).

**Video 1:** Prior to treatment, they exhibited generalized dystonia affecting all four limbs, the neck and face. This videoed attack lasted for several hours.

**Figure 1:**
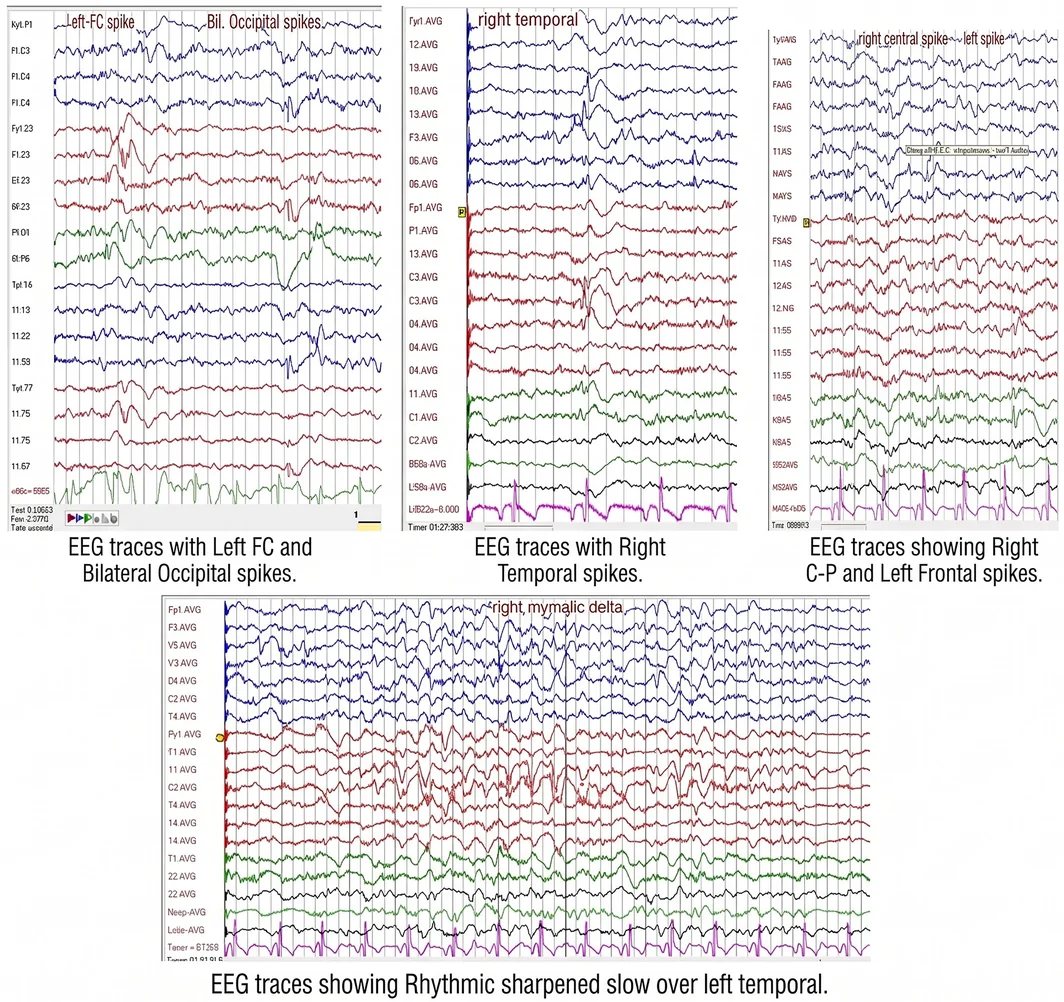
Intra‐event electroencephalography (EEG) recorded during episodes of dystonic limb posturing demonstrated a markedly abnormal background with severe encephalopathic slowing. Frequent multifocal epileptiform discharges were observed, predominantly arising from the right central and right frontal regions, indicating a significant epileptogenic tendency. No unequivocal electrographic seizures were identified during the recorded events. The EEG images were enhanced by Gemini owing to pixelation of original images making interpretation challenging.

These episodes were suspected generalized dystonia episodes; previous admissions for presumed status epilepticus without EEG correlates were retrospectively recognized as dystonic storm. The dystonia failed to improve with trihexyphenidyl, clonidine and clonazepam. The current medications on first review included levetiracetam, clonidine, diazepam and gabapentin. Vigabatrin was previously ineffective for seizure control and phenytoin at 10 mg twice daily in infancy for seizure management and discontinued due to lack of efficacy and febrile neutropenia.

Given the predicted GoF effect, lamotrigine, a presynaptic antagonist of voltage‐sensitive sodium channels, was initiated. This resulted in a dramatic reduction in dystonic episodes over 2 years with low‐dose lamotrigine (50 mg twice daily). Daily chloral hydrate use decreased to monthly and hospital admissions ceased. A recent breakthrough dystonic episode responded to dose escalation to 75 mg twice daily (Fig. 2).

**Figure 2:**
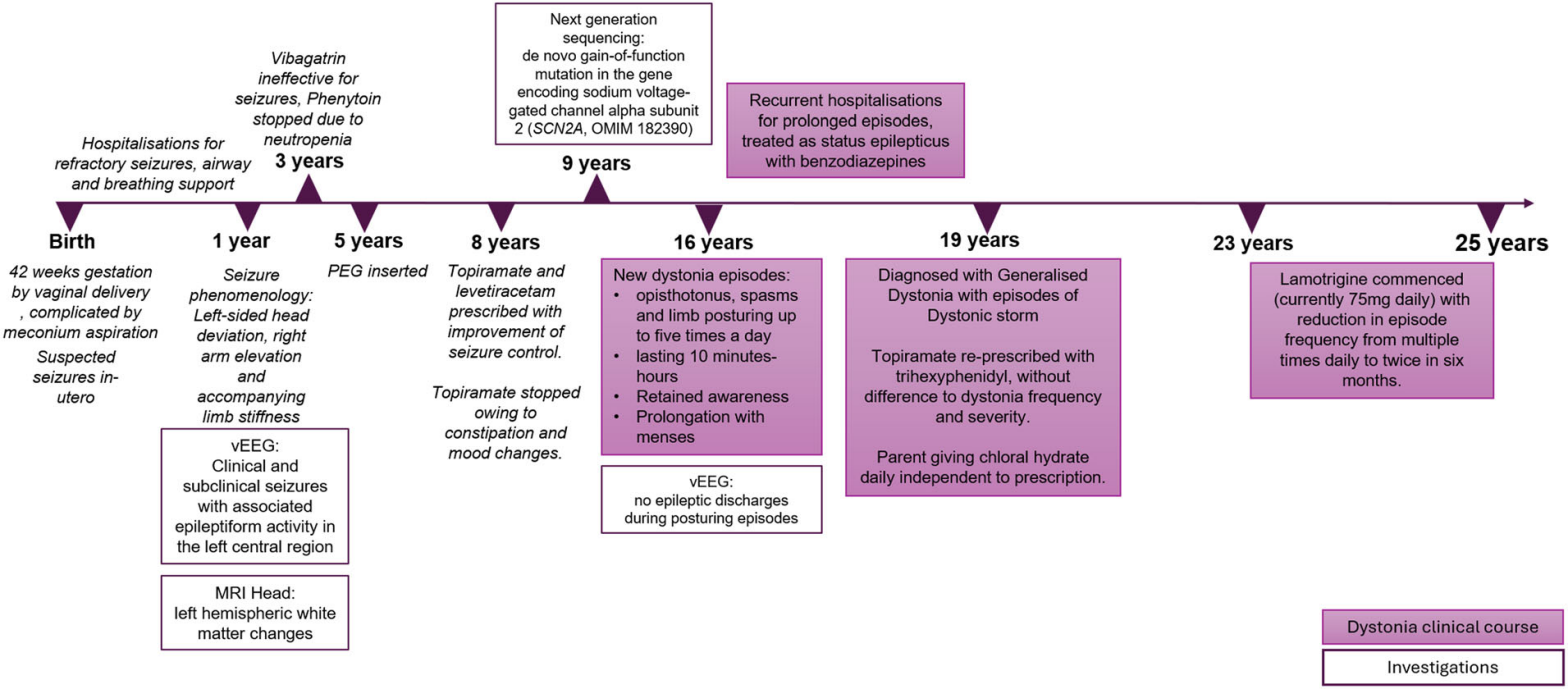
Clinical timeline, key milestones from clinical journey of epilepsy and dystonia management, respectively.

## Discussion

There is growing interest in monogenic epilepsy‐dyskinesia syndromes.^5^ *SCN2A* variants cause a spectrum of neurodevelopmental phenotypes ranging from self‐limited familial neonatal‐infantile epilepsy (SeLFNIE) to severe DEE.^3^, ^6^ SeLFNIE and *SCN2A*‐related DEE are usually caused by GoF variants, while loss‐of‐function (LoF) variants often lead to autism spectrum disorder (ASD) or intellectual disability, with or without later‐onset epilepsy. SeLFNIE presents with neonatal‐ or infantile‐onset seizures without cognitive impairment. *SCN2A*‐related DEE, typically due to de novo variants, includes early‐onset epilepsy and developmental delay. Now recognized to encompass a broad phenotypic spectrum, *SCN2A*‐related DEE includes comorbidities such as ASD and movement disorders. Over 90% present with their first seizure by 2 years of age. Hyperkinetic movement disorders, namely dystonia and chorea are observed, and this patient's dystonic phenotype was included in a previous review of monogenic epilepsy syndromes prior to treatment.^1^

This case highlights the value of precision medicine in guiding effective treatment. GoF mutations in *SCN2A* are described as responsive to sodium‐channel blockers, such as phenytoin and lamotrigine for seizure control. Their efficacy in treating associated hyperkinetic movement disorders remains poorly defined.^4^, ^7^, ^8^ In contrast, sodium‐channel blockers may be ineffective or detrimental for movement disorder phenotype in LoF channelopathies such as SCN1A‐related Dravet syndrome.^9^ Lamotrigine is a pre‐synaptic antagonist of voltage‐sensitive sodium channels and voltage‐gated calcium channels. Its mechanism of action includes pre‐synaptic membrane stabilization and enhanced GABAergic transmission.^10^ The authors acknowledge that the case is limited by the absence of functional studies and prior whole‐genome sequencing.

This case outlines the importance of investigating genotype–phenotype correlations and predicting functional effects of ion channel variants to employ precision treatment for movement disorders.

## Author Roles

(1) Research project: A. Conception, B. Organization, C. Execution; (2) Manuscript preparation: A. writing the first draft, B. Review and Critique.

I.S.P.: 1A, 1B, 1C, 2A, 2B.

P.M.: 1A, 1B, 1C, 2A, 2B.

S.S.: 1A, 1B, 1C, 2B.

T.T.W.: 1A, 1B, 1C, 2B.

## Disclosures

**Ethical Compliance Statement:** Consent has been received in written and verbal form from the patient's next of kin. This completed consent form has been stored securely within our institution. We confirm that we have read the Journal's position on issues involved in ethical publication and affirm that this work is consistent with those guidelines. This is a case report so was not subject to our institutional review board or ethics committee.

### Financial Disclosures and Conflicts of Interest

The authors declare that there are no conflicts of interest relevant to this work. The authors declare that there are no additional disclosures to report.

Author disclosures are available in the Supporting Information.

## Supporting information


**Data S1.** Supporting Information.

## Data Availability

Data sharing not applicable to this article as no datasets were generated or analysed during the current study.
